# Presence and Quantity of Botanical Ingredients With Purported Performance-Enhancing Properties in Sports Supplements

**DOI:** 10.1001/jamanetworkopen.2023.23879

**Published:** 2023-07-17

**Authors:** Pieter A. Cohen, Bharathi Avula, Kumar Katragunta, John C. Travis, Ikhlas Khan

**Affiliations:** 1 Department of Medicine, Cambridge Health Alliance, Somerville, Massachusetts; 2 Department of Medicine, Harvard Medical School, Boston, Massachusetts; 3 National Center for Natural Products Research, School of Pharmacy, University of Mississippi, University; 4 NSF International, Ann Harbor, Michigan

## Abstract

This case series study examines the accuracy of labels of dietary sports supplements containing botanical ingredients.

## Introduction

Since the US Food and Drug Administration (FDA) banned ephedra from dietary supplements in 2004, supplement manufacturers have promoted a complex variety of alternative botanical compounds for sports enhancement. Extracts of *Rauwolfia vomitoria *containing α-yohimbine, the caffeine-like compound methylliberine, the partial β_2_-agonist halostachine, the plant steroid turkesterone, and norepinephrine-like octopamine are all found in plants and are promoted in dietary supplements for their stimulant or anabolic effects.^1,2,3^

The FDA does not preapprove these ingredients, or any supplement ingredient, for either efficacy or safety before their introduction, but FDA inspections have found that supplement manufacturers often fail to comply with basic manufacturing standards, such as establishing the identity, purity, or composition of the final product. Given the products’ potentially complex physiologic effects and concerns regarding manufacturing quality, we determined the accuracy of dietary supplement labels declaring *R vomitoria*, methylliberine, halostachine, octopamine, and turkesterone.

## Methods

Dietary supplement products were included in this case series if they were labeled as containing 1 of the following ingredients: *R vomitoria*, methylliberine, turkesterone, halostachine, or octopamine. All products were purchased online, and products were excluded if the actual label did not list 1 of the 5 ingredients. Powder from the dietary supplement products was reconstituted in methanol and analyzed for the presence and quantity of the 5 ingredients and FDA-prohibited ingredients by liquid chromatography quadrupole time-of-flight mass spectrometry. See the eAppendix in Supplement 1 for additional details.

## Results

Of the 63 products purchased, 6 did not list 1 of the 5 ingredients on the label; therefore, 57 products were analyzed (13 listing *R vomitoria*; 21, methylliberine; 8, turkesterone; 7, halostachine; and 8, octopamine). Twenty-three of 57 products (40%) did not contain a detectable amount of the labeled ingredient. Of the products that contained detectable amounts of the listed ingredient, the actual quantity ranged from 0.02% to 334% of the labeled quantity (Table). Six of 57 products (11%) contained a quantity of the ingredient within 10% of the labeled quantity.

**Table.** Presence and Quantity of Botanical-Based Ingredients in Dietary Supplements

Product code	Claim on label	Labeled ingredient and amount per serving size	Measured ingredients per serving size, mean (SD), mg	Measured vs labeled ingredient per serving size, %	Prohibited ingredients detected	Prohibited ingredients per serving size, mean (SD), mg
*Rauwolfia vomitoria* products^a^						
A1	Nootropic, limitless energy	*R vomitoria* root-bark extract 2.5 mg	ND	0	Omberacetam (Noopept)^b^^,^^c^	5 (0.01)
B1	Fat burner	*R vomitoria* root extract	ND	NP	Octodrine^c^^,^^d^	319 (0.001)
C1	Thermogenic	*R vomitoria* extract (root bark)	ND	NP	ND	NA
D1	Preworkout	*R vomitoria* root extract	ND	NP	1,4-DMAA^e^^,^^f^	2.3 (0.01)
E1	Thermogen, precardio powerhouse, fat loss, energy, motivation	*R vomitoria* root-bark extract	ND	NP	ND	NA
F1	Preworkout	*R vomitoria* root extract 1.5 mg	ND	0	ND	NA
G1	Energy, aggression	*R vomitoria* extract (root-bark)	ND	NP	ND	NA
H1	Preworkout	*R vomitoria* root extract 1.5 mg	ND	0	ND	NA
I1	Preworkout	*R vomitoria* root extract 1.0 mg	ND	0	ND	NA
J1	No claim	*R vomitoria* root extract 1.0 mg	ND	0	ND	NA
K1	Preworkout	*R vomitoria* extract (root-bark) 225 µg	ND	0	ND	NA
L1	Weight loss, fat incinerator	*R vomitoria* extract (root-bark) 225 µg	ND	0	1,4-DMAA^e^^,^^f^	0.5 (0.01)
M1	Preworkout	*R vomitoria* extract (root-bark)	ND	NP	ND	NA
Methylliberine products						
N1	Thermo activator, energy, focus	Methylliberine 25 mg	33 (0.2)	133	ND	NA
O1	Energy, focus	Methylliberine	130 (0.2)	NP	ND	NA
P1	Metabolism optimization	Methylliberine 100 mg	125 (0.8)	125	ND	NA
Q1	Preworkout	Methylliberine 40 mg	52 (0.2)	129	ND	NA
R1	Preworkout	Methylliberine 100 mg	61 (0.3)	61	ND	NA
S1	Preworkout	Methylliberine 100 mg	136 (0.5)	136	ND	NA
T1	No claim	Methylliberine	16 (0.5)	NP	ND	NA
U1	Preworkout	Methylliberine 40 mg	59 (0.2)	147	ND	NA
V1	Energizing nootropic	Methylliberine 40 mg	134 (0.2)	334	ND	NA
W1	Preworkout	Methylliberine 25 mg	43 (0.02)	174	ND	NA
X1	Metabolic accelerator	Methylliberine 50 mg	69 (0.5)	138	ND	NA
Y1	Mind boosting nootropic	Methylliberine 75 mg	76 (0.3)	101	ND	NA
Z1	Fat burner	Methylliberine 100 mg	111 (0.3)	111	ND	NA
A2	Nootropic, mental concentration	Methylliberine 50 mg	68 (0.1)	136	ND	NA
B2	Metabolism booster	Methylliberine 25 mg	29 (0.6)	114	ND	NA
C2	Fast acting energy, mood, focus	Methylliberine 125 mg	133 (0.5)	106	ND	NA
D2	No claim	Methylliberine 40 mg	56 (0.2)	139	ND	NA
E2	No claim	Methylliberine	76 (0.2)	NP	ND	NA
F2	No claim	Methylliberine 150 mg	171 (0.1)	114	ND	NA
G2	No claim	Methylliberine 100 mg	115 (0.3)	115	ND	NA
H2	No claim	Methylliberine 100 mg	100 (0.1)	100	ND	NA
Turkesterone products^g^						
I2	Protein synthesis, enhance strength, increase lean mass	*Ajuga turkestanica* extract (10% turkesterone) 500 mg	0.01 (0.003)	0.02	ND	NA
J2	Bodybuilding	Turkesterone 500 mg	ND	0	ND	NA
K2	No claim	*A turkestanica* extract (turkesterone) whole plant	0.02 (0.004)	NP	ND	NA
L2	No claim	*A turkestanica* extract (10% turkesterone) 550 mg	ND	0	ND	NA
M2	No claim	*A turkestanica* extract (10% turkesterone) 500 mg	0.1 (0.0)	0.1	ND	NA
N2	Premium muscle growth	*A turkestanica* extract (standard minimum 10% turkesterone) 300 mg	0.03 (0.01)	0.1	ND	NA
O2	Testosterone anabolic support	*A turkestanica* extract (10% turkesterone)	ND	NP	ND	NA
P2	Bodybuilding	Turkesterone 500 mg	ND	0	ND	NA
Halostachine products^h^						
Q2	Intense, sustained thermogenic effect	Halostachine HCl	ND	NP	Deterenol^c^^,^^i^	0.02 (0.001)
					Oxilofrine^c^^,^^j^	0.2 (0.02)
					1,4-DMAA^e^^,^^f^	10 (0.1)
					Octodrine^d^^,^^f^	0.8 (0.02)
R2	Highly potent fat burner	Halostachine (*Halostachys capsica*) 40 mg	0.5 (0.1)	1.3	ND	NA
S2	Preworkout	Halostachine	2.0 (0.2)	NP	Deterenol^c^^,^^i^	0.01 (0.001)
T2	Stimulant	*H capsica* (aerial parts) extract 30 mg	ND	NP	ND	NA
U2	Preworkout	Halostachine 40 mg	1.0 (0.2)	3.0	Deterenol^c^^,^^i^	0.04 (0.001)
V2	Preworkout	Halostachine 100 mg	3.0 (0.1)	2.7	ND	NA
W2	Preworkout	*H capsica* (aerial parts) extract (standard to 80% halostachine) 100 mg	ND	0	ND	NA
Octopamine products^h^						
X2	Preworkout intensifier	Octopamine 30 mg	28 (0.1)	94.3	ND	NA
Y2	Preworkout	Octopamine	ND	NP	ND	NA
Z2	Nootropic brain fuel	Octopamine 30 mg	28 (0.2)	94	ND	NA
A3	Stimulant, weight loss	Octopamine	0.2 (0.1)	NP	ND	NA
B3	Fat loss	Octopamine	21 (0.1)	NP	ND	NA
C3	Weight management	Octopamine	ND	NP	ND	NA
D3	Appetite suppressor, thermogenic ignitor, fat incinerator	Octopamine	ND	NP	ND	NA
E3	Far burner	Octopamine 5 mg	6 (0.1)	110	ND	NA

Abbreviations: 1,4-DMAA, 1,4-dimethylamylamine; NA, not applicable; ND, not detected; NP, not possible (estimate not possible given lack of declared quantity of ingredient on label).

^a^ *R vomitoria* extract was considered not detected if analyses did not detect at least 5 common *R vomitoria* alkaloids (ie, rauwolsine, ajmaline, serpentinine, yohimbine, and reserpine), with a limit of detection of 10 ng/mL for each alkaloid.

^b^ The mechanism of action of omberacetam is unknown; it is marketed in Russia as Noopept. The drug has never been approved for use in the US.

^c^ Ingredient is declared on label.

^d^ Octodrine was marketed as part of a multicomponent oral medication in Germany. The drug has never been approved for oral use in the US.

^e^ 1,4-DMAA is an analogue of 1,3-dimethylamylamine, a sympathomimetic amine introduced in nasal inhalers in the 1940s in the US. 1,4-DMAA has never been approved as a medication for use in the US or elsewhere.

^f^ The ingredient was not declared on the label.

^g^ For turkesterone products, ND means not detected, with a limit of detection of 25 ng/mL.

^h^ For halostachine and octopamine products, ND means not detected, with a limit of detection of 50 ng/mL.

^i^ Deterenol is a β-agonist that was formerly available in Europe as an ophthalmologic preparation for the treatment of glaucoma. It has never been approved for use by the US Food and Drug Administration.

^j^ Oxilofrine is a pharmaceutical drug developed in Europe in the 1930s with cardiac stimulatory effects similar to ephedrine. The drug has never been approved for use by the US Food and Drug Administration.

Seven of 57 products (12%) were found to contain at least 1 FDA-prohibited ingredient (Table). Five different FDA-prohibited compounds were found, including 4 synthetic simulants, 1,4-dimethylamylamine, deterenol, octodrine, oxilofrine, and omberacetam. Six products contained 1 of these prohibited ingredients, and 1 product contained 4 different prohibited ingredients.

## Discussion

Eighty-nine percent of dietary supplement labels did not accurately declare the ingredients found in the products, and 12% of products contained FDA-prohibited ingredients. A prior study^4^ of dietary supplements, before the FDA ephedra ban, found that 6 of 12 products (50%) contained ephedra within 10% of the labeled amount. In a more recent study^5^ of caffeine content of sports supplements, 9 of 20 products (45%) contained a quantity of caffeine within 10% of the labeled quantity. In the current study, which to our knowledge is the first to quantify these 5 supplement ingredients, only 11% of products were accurately labeled and 5 different FDA-prohibited ingredients were found, including an unapproved drug available in Russia (ie, omberacetam), 3 drugs formerly available in Europe (ie, octodrine, oxilofrine, and deterenol), and 1 drug that has never been approved in any country (ie, 1,4-dimethylamylamine).^6^

The study has limitations, including that the sample size was small, only 1 sample of each brand was analyzed, and only supplements containing 1 of 5 targeted ingredients were analyzed. It is not known whether the results are generalizable to other botanical ingredients in sports supplements or whether quantities might also vary among batches within a given brand. Given these findings, clinicians should advise consumers that supplements listing botanical ingredients with purported stimulant or anabolic effects may not be accurately labeled and may contain FDA-prohibited drugs.

## Notes

Supplement 1. **eAppendix.** Supplemental Methods
eReferences

Supplement 2.
Data Sharing Statement
