# Supplementary material for: Presence and Quantity of Botanical Ingredients With Purported Performance-Enhancing Properties in Sports Supplements
Source: JAMA Netw Open. 2023 Jul 17;6(7):e2323879. doi: 10.1001/jamanetworkopen.2023.23879 (PMC10352857; doi:10.1001/jamanetworkopen.2023.23879)
Supplement: Supplement 1. — eAppendix. Supplemental Methods eReferences [file jamanetwopen-e2323879-s001.pdf]

Supplemental Online Content

Cohen PA, Avula B, Katragunta K, Travis JC, Khan I. Presence and quantity of botanical ingredients with purported performance-enhancing properties in sports supplements. *JAMA Netw Open*. 2023;6(7):e2323879. doi:10.1001/jamanetworkopen.2023.23879

eAppendix. Supplemental Methods

eReferences

This supplemental material has been provided by the authors to give readers additional information about their work.

© 2023 Cohen PA et al. *JAMA Network Open.*

eAppendix. Supplemental Methods

1. Chemicals and reagents

High-performance liquid chromatography (HPLC) grade acetonitrile and formic acid were purchased from Fisher Scientific (Fair Lawn, NJ, USA). Water was obtained using a milliQ-Gradient system (Millipore, Billerica, USA). The standard compounds, turkesterone, octopamine, halostachine, yohimbine, ajmaline, serpentinine, reserpine, 1,4-dimethylamylamine (1,4-DMAA), octodrine (1,5-DMHA) and omberacetam (Noopept) were purchased from Sigma (St. Louis, MO, USA) (> 99 % purity). Methylliberine (purity > 99 %) was purchased from LabNetwork (Saint Paul, MN, USA) and rauwolscine (purity > 99 %) was purchased from Indofine Chemical Company, Inc. (Hillsborough, NJ, US).

2. *Rauwolfia vomitoria* Analysis

2.1 Dietary supplements were included if they: a) had been entered into the National Institutes of Health's Dietary Supplement Label Database in the year 2020, and b) were found searching the database for *Rauwolfia vomitoria* in August, 2021. Supplements entered into the database only in 2020 were used because more than 200 supplements listing *Rauwolfia vomitoria* exist in the database and it was impractical to purchase them all. Supplements were purchased online in September, 2021. After purchase, the label was inspected and supplements were excluded if the product did not contain the terms "dietary supplement" and ingredient "Rauwolfia vomitoria" on the actual label. Sixteen brands of supplements were identified, 2 were unavailable for purchase, and 1 did not include "Rauwolfia vomitoria" on the actual label; therefore, 13 *Rauwolfia vomitoria* products were analyzed in April, 2022. Data processed using MassHunter Qualitative version B.07.00 (Agilent Technologies, Santa Clara, CA, USA) and data calculations were performed using Microsoft 365 Excel software (Microsoft Corporation, Redmond, WA, USA) in April, 2022.

2.2 Preparation of the purchased supplements (capsules/tablets/powders)

The dietary supplements were in the form of either capsules, tablets or powders. For powders, 5 grams were weighed, ground and uniformly mixed; for capsules, 5 capsules and tablets were weighed, opened and their contents were mixed and triturated in a mortar and pestle. Next, about 1000 mg for powders and the average weight of capsule content or tablets of the homogenized samples were weighed in duplicate into centrifuge tubes, resuspended in 2.5 mL of methanol and sonicated for 30 minutes. The mixture was centrifuged for 15 minutes at 959 × g. The supernatant, consisting of a clear solution, was transferred to a 10 mL volumetric flask. The procedure was repeated three times, combining the supernatants. The solution was brought a final volume of 10 mL with methanol and mixed thoroughly. All samples were filtered through a 0.45 µm polytetrafluoroethylene (PTFE) membrane filter prior to injection.

2.3 Instrumental conditions

Liquid Chromatography-Quadrupole Time-of-Flight Mass Spectrometry (LC-QToF-MS)

The liquid chromatography was performed on an Agilent Series 1290 (Agilent Technologies, Santa Clara, CA, USA) coupled to a QToF-MS detector (Model #G6530A, Agilent Technologies, Santa Clara, CA, USA) equipped with an electrospray ionization (ESI) source with Jet Stream technology. The chromatographic separation was achieved on an Agilent Poroshell 120 EC-C_{18} (2.1 × 150 mm, 2.7 µm) column. The mobile phase consisted of 0.1 % formic acid in water (A) and 0.1 % formic acid in acetonitrile (B). The flow was set at 0.21 mL/min and the temperature of the column was set at 35 °C. The following gradient elution method was utilized: an initial 5

© 2023 Cohen PA et al. *JAMA Network Open.*

minutes equilibration with 5 % B, increased to 30 % B in 20 minutes, and a 3 minutes wash with 100 % B as the final step. The mass spectrometer (MS) was operated with the following parameters: drying gas (nitrogen, N_2) flow rate was set at 11.0 L/min; drying gas temperature, 325 °C; nebulizer, 30 psig; sheath gas temperature, 300 °C; sheath gas flow, 11 L/min; capillary, 3500 V; skimmer, 65 V; OCT 1 RF V_{pp}, 750 V and fragmentor voltage, 100 V. The acquisition was controlled by Agilent MassHunter Acquisition Software Ver. A.05.01, and the spectra were collected in positive ion mode scanning over the range of *m/z* 100 –1100 (MassHunter Qualitative software Ver. B.10.00). MS-MS spectra were generated by collision-induced dissociation (CID) of the metabolite ions at 40 eV. Accurate mass measurements were obtained using ion correction techniques with reference masses at *m/z* 121.0509 (protonated purine) and 922.0098 [protonated hexakis (1H, 1H, 3H-tetrafluoropropoxy) phosphazine or HP-921] in positive ion mode. The identification of *Rauwolfia vomitoria* in the products was determined by the presence of seven prominent alkaloids (i.e. rauwolsine, ajmaline, 17-*O*-acetyl ajmaline, serpentinine, rauvanine, yohimbine and reserpine). We define the positive identification of *Rauwolfia vomitoria* if all these seven alkaloids were present. Rauwolsine and yohimbine were observed in some of the dietary supplements, indicating the addition of synthetic or isolated compounds.

3. Methylliberine products

3.1 Methylliberine products were included if they were either a) found using the Google Images search engine searching for "dietary supplement" and "methylliberine" or b) if they were in the National Institute of Health's Dietary Supplement Label Database as listing methylliberine as an ingredient and product labels provided online also included methylliberine as an ingredient in June, 2022. The Google Images search engine was used to capture "methylliberine" listed on the product label. All supplements were purchased online in September 2022. Twenty-six products were identified in the search, and 5 products were either discontinued or out-of-stock; 21 products were analyzed in October, 2022. The data processed using Empower v3.7.0 software (Waters Corporation, Milford, MA, USA) in October, 2022, and data calculations were performed using Microsoft 365 Excel software (Microsoft Corporation, Redmond, WA, USA) in October, 2022.

3.2 Preparation of reference materials and samples

Preparation of reference standard solutions

A stock solution of methylliberine was prepared at a concentration of 1 mg/mL in methanol. The calibration was prepared in methanol at five different concentrations, ranging from 5 to 250 µg/mL.

Preparation of the purchased supplements (capsules/powders)

The dietary supplements were in the form of either capsules or powders. For powders, 5 grams were weighed, ground and uniformly mixed; for capsules, 5 capsules were weighed, opened and their contents were mixed and triturated in a mortar and pestle. Next, about 100 mg of the homogenized samples were weighed in duplicate into centrifuge tubes, resuspended in 2.5 mL of methanol and sonicated for 30 minutes. The mixture was centrifuged for 15 minutes at 959 × g. The supernatant, consisting of a clear solution, was transferred to a 10 mL volumetric flask. The procedure was repeated three times combining the supernatants. The solution was brought a final volume of 10 mL with methanol and mixed thoroughly. All samples were filtered through a 0.45 µm PTFE membrane filter prior to injection.

© 2023 Cohen PA et al. *JAMA Network Open.*

3.3 Instrumental conditions

Ultra-High-Performance Liquid Chromatography-Photodiode Array (UHPLC-PDA) Analysis

All analyses were performed on a Waters Acquity UPLC™ H-Class system (Waters Corp., Milford, MA, USA) including quaternary solvent manager, sampler manager-flow through needle, column heater, and photo-diode array (PDA) detector connected to Waters Empower 3.7.0 data station. An Acquity UPLC™ BEH Shield RP18 column (100 mm × 2.1 mm I.D., 1.7 µm), also from Waters, was used. The column and sample temperature were maintained at 40 °C and 15 °C, respectively. The column was equipped with a LC-18 guard column (Vanguard 2.1 × 5 mm, Waters Corp., Milford, MA, USA). The mobile phase consisted of water (0.1 % formic acid) (A), acetonitrile (B) (0.1 % formic acid) at a flow rate of 0.23 mL/min, which were applied in the following linear gradient elution: 0-5 min, 10 % B to 40 % B. Separation was followed by a 2-minute column washing with 100 % B and a re-equilibration period of 4.5 minutes at the starting conditions. A strong needle wash solution (95/5; acetonitrile/water) and a weak needle wash solution (10/90; acetonitrile/water) were used. All solutions were filtered via 0.45 µm membrane filters and degassed prior to use. The total run time for analysis was 3 minutes. The injection volume was 2 µL. The detection wavelength for methylliberine was 284 nm. Peak identity for methylliberine was assigned by analysis of a refence standard and samples fortified with the reference standard, comparing their retention times and ultra-violet absorbance spectra.

3.4 Validation procedure

The newly developed UHPLC-PDA method was validated with respect to selectivity, sensitivity, the limit of detection (LOD), the limit of quantification (LOQ), stability, precision, accuracy, specificity and linearity according to International Council for Harmonisation (ICH) guidelines [1].

The specificity of the method was conducted by comparing chromatograms of blank (methanol, matrix without methylliberine) products and spiked blanks (methylliberine added to methanol and brand code R1, B2). A comparison of methanol blanks with samples and spiked samples (methylliberine added to methanol or matrix solution) demonstrated the specificity and selectivity of the used methodology.

The LOD and LOQ were determined by injecting a series of dilute solutions with known concentrations for each standard. LOD and LOQ were assigned at the concentrations where the signal-to-noise ratio equaled 3 and 10, respectively. A five-point calibration for methylliberine showed a linear correlation between concentration and peak area. Calibration data indicated the linearity ($r^2 > 0.99$) of the detector response. The limits of detection and limits of quantification were found to be 100 and 300 ng/mL, respectively. All samples and standard solutions were injected in triplicate.

The accuracy of the assay method was evaluated by spiking two products (product code R1, B2) in duplicate using concentration levels of 5.0 and 100 µg/mL. The accuracy of the method was determined for the related compound by spiking sample (product code R1, B2) with a known amount of methylliberine standard. The percentage recovery of these samples ranged from 97 – 105 %.

Precision of a method is the degree of agreement among individual analytical results when the procedure is applied repeatedly to multiple samples of each product. The intra- and inter-day precision were estimated by analyzing multiple replicates of two products (product code R1, B2). The intra-day precision of the assay was estimated by calculating the relative standard deviation (RSD) for the analysis of samples in three replicates (n=3) of each product and inter-day precision was determined by the analysis of three replicates each of the same product on three consecutive

© 2023 Cohen PA et al. *JAMA Network Open.*

days. The intra-day RSD for the replicates were between 1.0 and 2.5 % and RSD for the day to day replicates was 0.2 - 1.1 % [precision as relative standard deviation (% RSD) was calculated as % RSD = SD / mean × 100 from the calculated standard deviation (SD) and mean values].

The sample solution (brand code N1, R1, B2, G2) and standard solutions (50 µg/mL and 100 µg/mL) were prepared as per the proposed method and subjected to stability study at room temperature for 72 hours. The sample solution was analyzed at initial and at three-time intervals up to 72 hours. No significant changes were observed in the concentrations of the components analyzed with respect to time.

4. Turkesterone products

4.1 Turkesterone products were selected by searching the Natural Medicines Comprehensive Database (https://naturalmedicines.therapeuticresearch.com) for turkesterone supplements in May 2022. Ten products were identified. Two products were not available for sale online in May 2022; therefore, 8 products were analyzed in June, 2022. The data processed using MassHunter Qualitative version B.07.00 (Agilent Technologies, Santa Clara, CA, USA) in June, 2022, and data calculations were performed using Microsoft 365 Excel software (Microsoft Corporation, Redmond, WA, USA) in June 2022.

4.2 Preparation of reference materials and samples

Preparation of reference standard solutions

A stock solution of the turkesterone was prepared at a concentration of 1 mg/mL in methanol. The calibration was prepared in methanol at five different concentrations, ranging from 0.1 to 5 µg/mL.

Preparation of the purchased supplements (capsules/powders)

The dietary supplements were in the form of either capsules or powders. For powders, 5 grams were weighed, ground and uniformly mixed; for capsules, 5 capsules were weighed, opened and their contents were mixed and triturated with a mortar and pestle. Two homogenized samples were weighed for each product separately into centrifuge tubes and suspended in 2.5 mL of methanol, one in duplicate at 1000 mg for powders and one in duplicate at the average weight per capsule. The mixture was sonicated for 30 minutes followed by centrifugation for 15 minutes at 959 × g. The supernatant, consisting of a clear solution, was transferred to a 10 mL volumetric flask. The procedure was repeated three times combining the supernatants. The solution was brought to a final volume of 10 mL with methanol and mixed thoroughly. All samples were filtered through a 0.45µm PTFE membrane filter prior to injection.

4.3 Instrumental conditions

Liquid Chromatography-Quadrupole Time of Flight Mass Spectrometry (LC-QToF-MS)

The liquid chromatography was performed on an Agilent Series 1290 (Agilent Technologies, Santa Clara, CA, USA) coupled to a QToF-MS detector (Model #G6530A, Agilent Technologies, Santa Clara, CA, USA) equipped with an electrospray ionization (ESI) source with Jet Stream technology. The chromatographic separation was achieved on an Agilent Poroshell 120 EC-C_{18} (2.1 × 150 mm, 2.7 µm) column. The mobile phase consisted of 0.1 % formic acid in water (A) and 0.1 % formic acid in acetonitrile (B). The flow was set at 0.21 mL/min and the column temperature was set at 40 °C. The following elution methodology was utilized: a 5-minute equilibration with 5% B, followed by a gradient elution to 40% B in 15 minutes and in the next 5 minutes to 100% B, with a 3-minute wash with 100% B as the final step. The mass spectrometer (MS) was operated in positive mode with the following parameters: drying gas (N_2) flow rate was

set at 11.0 L/min; drying gas temperature, 325 °C; nebulizer, 30 psig, sheath gas temperature, 300 °C; sheath gas flow, 11 L/min; capillary, 3500 V; skimmer, 65 V; OCT 1 RF V_{pp}, 750 V and fragmentor voltage, 150 V. The acquisition was controlled by Agilent MassHunter Acquisition Software Ver. A.05.01, and the spectra collected in scanning mode over the range of 50-1200 *m/z* (MassHunter Qualitative software Ver. B.10.00) in positive ion mode. MS-MS spectra were generated by collision-induced dissociation (CID) at 40 eV. Accurate mass measurements were obtained using ion correction techniques with reference masses at *m/z* 121.0509 (protonated purine) and 922.0098 [protonated hexakis (1H, 1H, 3H-tetrafluoropropoxy) phosphazine or HP-921] in positive ion mode

4.4 Validation procedure

The newly developed LC-QToF method was validated with respect to selectivity, sensitivity, limit of detection (LOD), limit of quantification (LOQ), stability, precision, accuracy, specificity and linearity according to International Council for Harmonization (ICH) guidelines [1].

High resolution mass spectrometry (>20,000 resolving power) was used for the detection of turkesterone ($C_{27}H_{44}O_8$). An extracted ion chromatogram (*m/z* 497.3109 with an error tolerance of 2 ppm) for the target analyte was used for quantification. Retention time in the extracted ion chromatogram, mass accuracy and the fragmentation pattern of the compound in the samples were matched with those from a turkesterone reference standard for reliable identification. The specificity of the method was conducted by comparing chromatograms of blank (methanol) with products and spiked blanks (turkesterone added to methanol and product code L2). A comparison of methanol blanks with samples and spiked blanks (turkesterone added to methanol or matrix solution) demonstrated the specificity and selectivity of the used methodology.

The LOD and LOQ were determined by injecting a series of dilute solutions with known concentrations for each standard. LOD and LOQ were assigned at the concentrations where the signal-to-noise ratio equaled 3 and 10, respectively. A five-point calibration for turkesterone showed a linear correlation between concentration and peak area. Calibration data indicated the linearity ($r^2 > 0.99$) of the detector response. The limits of detection and limits of quantification were found to be 25 and 100 ng/mL, respectively. All samples and standard solutions were injected in triplicate.

The accuracy of the assay method was evaluated by spiking one product (product code L2) in duplicate at concentration levels of 0.1, 2.0 and 5 µg/mL for turkesterone. These samples spiked with known amounts of the standard compound mixture were extracted as mentioned under optimized conditions. The percentage recovery of these samples ranged from 96 – 101 % for turkesterone.

The intra- and inter-day precision were estimated by analyzing multiple replicates of two products (product code I2, M2). The intra-day precision of the assay was estimated by calculating the relative standard deviation (RSD) for the analysis of samples in three replicates (n=3) of each product and inter-day precision was determined by the analysis of three replicates each of same product on three consecutive days. The intra-day RSD for the replicates were between 0.04 and 0.1 % and RSD for the day to day replicates was 0.1 %.

The sample solution (product code I2, M2) and standard solutions (1 µg/mL and 5 µg/mL) were prepared as per the proposed method and subjected to stability study at room temperature for 72 hours. The sample solution was analyzed at initial and at three-time intervals up to 72 hours. No significant changes were observed in the concentrations of the components analyzed with respect to time.

5. Halostachine and octopamine products

5.1 To select the halostachine products: the Google Images search engine was used to search for "dietary supplement" and "halostachine" in May, 2022. The Google Images search engine was used to capture "halostachine" listed on the product label. The first 20 products, excluding advertisements, found using this search were purchased online in May, 2022; when the products were purchased online in May, 2022, 8 products had been discontinued or were out of stock and 12 products arrived. Upon inspection of the label on the actual products, 5 products did not list halostachine and were excluded from the study; therefore, 7 halostachine dietary supplements were analyzed from June, 2022 to July, 2022. The data processed using MassHunter Qual version B.07.00 (Agilent Technologies, Santa Clara, CA, USA) in July, 2022, and data calculations were performed using Microsoft 365 Excel software (Microsoft Corporation, Redmond, WA, USA) in July, 2022.

To select the octopamine products: supplements were included if they were in the National Institute of Health's Dietary Supplement Label Database as listing octopamine as an ingredient and product labels provided online also included octopamine as an ingredient in June, 2022. Thirteen products met the inclusion criteria. All supplements were purchased online in September, 2022. Five products were discontinued or out-of-stock, therefore 8 octopamine supplements were analyzed in October, 2022. The data processed using MassHunter Qual version B.07.00 (Agilent Technologies, Santa Clara, CA, USA) in October, 2022, and data calculations were performed using Microsoft 365 Excel software (Microsoft Corporation, Redmond, WA, USA) in October, 2022.

5.2 Preparation of reference materials and samples

Preparation of reference standard solutions

A stock solution of the reference compound was prepared at a concentration of 1 mg/mL in methanol. The calibration was prepared in methanol at five different concentrations, ranging from 0.05 to 10 µg/mL.

Preparation of the purchased supplements (capsules/powders)

The dietary supplements were in the form of either capsules or powders. For powders, 5 grams were weighed, ground and uniformly mixed; for capsules, 5 capsules and tablets were weighed, opened and their contents were mixed and triturated in a mortar and pestle. Next, about 100 mg of the homogenized samples were weighed in duplicate into centrifuge tubes and resuspended in 2.5 mL of methanol. The mixture was sonicated for 30 minutes followed by centrifugation for 10 minutes at 959 × g. The supernatant, consisting of a clear solution, was transferred to a 10 mL volumetric flask. The procedure was repeated three times and the supernatants were combined. The solution was brought to a final volume of 10 mL with methanol and mixed thoroughly. All samples were filtered through a 0.45 µm PTFE membrane filter prior to injection.

5.3 Instrumental conditions

Liquid Chromatography-Quadrupole Time of Flight Mass Spectrometry (LC-QToF-MS)

The liquid chromatography was performed on an Agilent Series 1290 (Agilent Technologies, Santa Clara, CA, USA) coupled to a QToF-MS detector (Model #G6530A, Agilent Technologies, Santa Clara, CA, USA) equipped with an electrospray ionization (ESI) source. The instrumental conditions were same as in the reported method [2].

© 2023 Cohen PA et al. *JAMA Network Open.*

5.4 Validation procedure

Validation procedure was same as in the reported method [2].

6. Identification and quantification of other prohibited ingredients from dietary supplements

Sample preparation was the same as described above, and instrumental conditions were the same as in the reported method [2]. The supplements were screened for synthetic or hidden compounds using Agilent MassHunter Forensics and Toxicology (9203 compounds) Personal Compound Database (PCD). During this screening process, 1,4-DMAA, oxilofrine (methylsynephrine), deterenol (isopropylnorsynephrine or isopropyloctopamine), octodrine (1,5-DMHA) and omberacetam (Noopept) were detected in various supplements (Table) and were confirmed as well as quantified using the reference standards. The screening for synthetic and hidden compounds were performed using MassHunter PCDL Manager version B.08.00 (Agilent Technologies, Santa Clara, CA, USA) in November, 2022.

eReferences

[1] ICH. Validation of Analytical Procedures: Text and Methodology. ICH Harmonized Tripartite Guidelines Nov. 2005

[2] Avula B, Bae JY, Chittiboyina AG, Wang YH, Wang M, Khan IA. Liquid chromatography-quadrupole time of flight mass spectrometric method for targeted analysis of 111 nitrogen-based compounds in weight loss and ergogenic supplements. J Pharm Biomed Anal. 2019 Sep 10;174:305-323. doi: 10.1016/j.jpba.2019.05.066. Epub 2019 May 31.

© 2023 Cohen PA et al. *JAMA Network Open.*
