# Supplementary material for: Presence and Quantity of Botanical Ingredients With Purported Performance-Enhancing Properties in Sports Supplements
Source: JAMA Netw Open. 2023 Jul 17;6(7):e2323879. doi: 10.1001/jamanetworkopen.2023.23879 (PMC10352857; doi:10.1001/jamanetworkopen.2023.23879)
Supplement: Supplement 2. — Data Sharing Statement [file jamanetwopen-e2323879-s002.pdf]

Data Sharing Statement

Cohen. Presence and Quantity of Botanical Ingredients With Purported Performance-Enhancing Properties in Sports Supplements. *JAMA Netw Open*. Published July 17, 2023. doi:10.1001/jamanetworkopen.2023.23879

Data
Data available: No

Additional Information
Explanation for why data not available: Not applicable.
